# Chemical Composition, Edible Safety, and Antioxidant Activity Evaluation of Flowers in a Medicinal Plant *Dendrobium chrysotoxum*

**DOI:** 10.1002/fsn3.70067

**Published:** 2025-04-18

**Authors:** Xu Susu, Li Jinwei, Pu Ran, Xiong Wenyan, Guan Yanhui, Ma Suyun, Liang Zhengwei, Zhang Jingli, Chen Junwen

**Affiliations:** ^1^ College of Agronomy and Biotechnology Yunnan Agricultural University Kunming China; ^2^ National & Local Joint Engineering Research Center on Germplasm Innovation Yunnan Agricultural University Kunming China; ^3^ Utilization of Chinese Medicinal Materials in Southwestern China, The Key Laboratory of Medicinal Plant Biology of Yunnan Province Yunnan Agricultural University Kunming China; ^4^ Yunnan Institute of Tropical Crops Kunming China; ^5^ Flavor Beverage Institute Chinese Academy of Tropical Agriculture Science Haikou China

**Keywords:** antioxidant capacity, *Dendrobium chrysotoxum*, edible flowers, toxicological safety assessment, widely targeted metabolomic

## Abstract

*Dendrobium chrysotoxum* stem is a famous traditional ethnic medicinal, and its flowers have the potential to be used as new food or traditional Chinese medicine resources. However, the chemical composition, edible safety, and antioxidant capacity of *D. chrysotoxum* flowers are insufficient. In this study, we collected 49 samples of *D. chrysotoxum* flowers from 49 cultivation sites across five southern provinces in China. The heavy metals and pesticide residues in *D. chrysotoxum* flowers were determined. Furthermore, this experiment conducted a systematic toxicological safety assessment, analyzed the chemical composition, and evaluated the antioxidant capacity of *D. chrysotoxum* flowers. The results showed that none of the 49 samples of *D. chrysotoxum* flowers posed safety risks regarding the five heavy metals and 33 banned pesticide residues. The toxicological safety assessment confirmed that there was no observed toxicity in mice at an acute oral dose of 30.0 g kg^−1^. The *D. chrysotoxum* flower extract showed no mutagenic effects on mice. Within the dosage range of 6.67 g kg^−1^, the *D. chrysotoxum* flower extract did not induce micronuclei in mouse bone marrow erythrocytes or cause chromosomal aberrations in mouse spermatocytes. Additionally, within the dosage range of 10 g kg^−1^, the *D. chrysotoxum* flower extract exhibited no subchronic toxicity in mice. A total of 2,047 metabolites were identified in *D. chrysotoxum* flowers and classified into 15 superclasses using a widely targeted metabolomics method. Sixteen amino acids were measured using high‐performance liquid chromatography. This study demonstrates that *D. chrysotoxum* flowers are safe for human consumption at recommended dosages. Furthermore, *D. chrysotoxum* flowers contain a variety of functional compounds with antioxidant properties, making them suitable for development and use as edible flowers.

## Graphical abstract

This manuscript presents an analysis of heavy metals and pesticide residues in 49 samples of *Dendrobium chrysotoxum* flowers collected from five provinces in southern China. A randomly selected sample of *D. chrysotoxum* underwent a systematic toxicological safety assessment, including an analysis of its chemical composition and an evaluation of its antioxidant capacity.

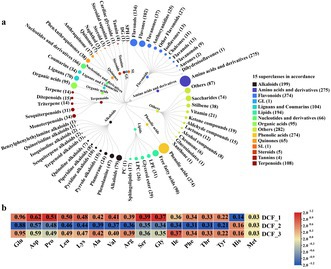

## 1 Introduction

*Dendrobium* is the second largest genus in the Orchidaceae, with 78 reported species, mainly distributed in Asian and European countries (Hu et al. 2023). Dendrobium orchids are highly regarded for their vibrant colors, long‐lasting blooms, and pleasant fragrance, all of which contribute to their ornamental appeal. Ongoing studies on *Dendrobium* have revealed the presence of numerous chemical components that offer health benefits to humans. The traditional uses of *Dendrobium* include treatments for dermatologic disorders, metabolic syndromes, nervous system disorders, and musculoskeletal system disorders (Li et al. 2023). Modern pharmacological studies have shown that *Dendrobium* has various pharmacological effects, including antitumor, antifatigue, antioxidant, anti‐aging, anti‐inflammatory, liver protection, gastrointestinal protection, antidiabetic, etc. (Chen et al. 2021; Li et al. 2023; Wang 2021). The pharmacological activity of *Dendrobium* is related to its rich content of alkaloids, polysaccharides, bibenzyls, phenanthrene compounds, phenolic compounds, and sesquiterpenoids (Li et al. 2023; Yang et al. 2023). The chemical composition and pharmacological activity of different organs of *Dendrobium* species have always been the main focus of *Dendrobium* research, aiming to develop natural medicines and supplementary agents with minimal side effects for patients.

Currently, the *Dendrobium* species most widely cultivated are *D. officinale* , *D. huoshanense*, *D. chrysotoxum*, *D. nobile*, and *D. moniliforme* (Wang 2021). *D. chrysotoxum* is mainly distributed in Yunnan, China, northeastern India, and Southeast Asia, with a planting area of about 4000 ha in China (Hu et al. 2023). In the Chinese Pharmacopoeia, only the stems of *Dendrobium* are permitted as medicinal materials (Wang 2021). Therefore, in the production of medicinal *Dendrobium*, the leaves and flowers are usually discarded (Wang 2021). Currently, the cultivation area of *Dendrobium* in China exceeds 30,000 ha (Yang et al. 2022), with nearly 4000 ha dedicated to *D. chrysotoxum*, producing around 1200 t of fresh flowers (Hu et al. 2023). Utilizing the flowers of *D. chrysotoxum* can not only reduce resource waste but also improve economic efficiency (Hu et al. 2023).

China has a long history of consuming edible *Dendrobium* flowers, and currently, the flowers of *D. officinale* are consumed through various methods, including brewing tea, soaking in alcohol, stewing soup, beverages, salads, candies, and jellies (Cheng et al. 2019; Ketsa and Warrington 2023; Zhang et al. 2019; Zhao et al. 2021). They can also serve as raw materials for pharmaceutical products and cosmetics (Kanlayavattanakul et al. 2018). Furthermore, jelly made primarily from these flowers meets food hygiene and safety standards through microbiological testing (Yang et al. 2021). *D. chrysotoxum* flowers have the potential to be used as a traditional Chinese medicine or new food. However, current literature lacks further reports on the application of *D. chrysotoxum* flowers as food.

In recent years, studies have found that the leaves and flowers of *D. huoshanense* (Liu et al. 2020) and *D. officinale* (Wen et al. 2021) have similar chemical compositions and pharmacological activities to the stems. Like the stem, the flowers of *D. officinale* also contain various antioxidant substances such as anthocyanins (Zhang et al. 2018), total flavonoids (Miao et al. 2019a), and polysaccharides (Miao et al. 2019b; Nie et al. 2022). There are few reports on the chemical composition of *D. chrysotoxum* flowers. Huang found that *D. officinale* , *D. nobile*, *D. thyrsiflorum*, and *D. chrysotoxum* flowers all have high levels of polysaccharides, total flavonoids, and amino acids (Huang et al. 2017). Robustelli analyzed the main components of the essential oil from *D. chrysotoxum* flowers originating from Degerndorf, Germany, and the components include esters, saturated hydrocarbons, oxygenated terpenes, alcohols, aldehydes, and terpenes (Robustelli et al. 2021). Hu reported on the types and contents of flavonoids in the dried flower extracts of *D. chrysotoxum* from Baoshan City, China (Hu et al. 2023). Further research is needed on the types and contents of chemical components in *D. chrysotoxum* flowers. Modern pharmacological studies have shown that *D. officinale* flowers can alleviate alcoholic liver injury in mice (Liang et al. 2018) and lower blood pressure in rats with hypertension caused by high sugar and high‐fat alcohol consumption (Yang et al. 2024). China has initiated trials in several provinces to develop the stems, leaves, and flowers of *Dendrobium* as local specialty new food ingredients and health products (Yang et al. 2022). The stems, leaves, and flowers of *D. officinale* were approved by the National Health Commission of China as new food ingredients in 2013, 2017, and 2018, respectively (Cheng et al. 2019; Zhang et al. 2019). The safety of *D. officinale* has been extensively studied (Chen et al. 2021). The acute toxicity test (12.0 g kg^−1^), genetic toxicity tests, and 90‐day feeding test (1.08, 1.67, and 5.00 g kg^−1^) in rats indicated that *D. officinale* showed no noticeable signs of toxicity, genetic toxicity, or mutagenicity within the tested dose ranges (Li et al. 2019). Likewise, oral administration with the leaves and flowers of *D. officinale* at the doses of 0, 2.0, 4.0, and 6.4 g kg^−1^ for 90 days did not exhibit apparent adverse effects on sperm quality and testicular tissue morphology in parent and offspring rats (Fu et al. 2017; Fu et al. 2020). The toxicological evaluation of *D. taiseed tosnobile* and *D. moniliforme* stems has also been reported (Lee et al. 2016; Yang et al. 2018). However, there is currently a gap in the safe evaluation of the stems, leaves, and flowers of *D. chrysotoxum—while* studies on heavy metals and pesticide residues in *Dendrobium* mainly focus on stems and leaves, reports on heavy metals and pesticide residues in *Dendrobium* flowers are rare (Liu et al. 2023; Zhang et al. 2022).

In order to quantitatively analyze the chemical composition of *D. chrysotoxum* flowers and fill the gap in the safety evaluation of *D. chrysotoxum* flowers. This study collected 49 samples of *D. chrysotoxum* flowers from the main cultivation sites in China and detected the heavy metal and banned pesticide residue levels in each sample. Moreover, the toxicity evaluation, metabolomics analysis, and antioxidant capacity evaluation of the *D. chrysotoxum* flowers were conducted. The results of this paper provide a theoretical basis for *D. chrysotoxum* flowers as nontoxic and multifunctional new food ingredients.

## 2 Materials and Methods

### 2.1 Materials and Reagents

A total of 49 flower samples were collected in May 2022 from two‐year‐old artificially cultivated *D. chrysotoxum* at representative cultivation sites across China (Table 1). standardized collection procedures were strictly followed during the sampling process. Specifically, the timing of collection was chosen during the peak flowering period to ensure the representativeness and quality of the collected samples. Additionally, sterile tools were used for collection to avoid contamination during the sampling process. After collection, the samples were immediately transported and stored appropriately to maintain their freshness and integrity. Each sample was assigned a unique specimen number for identification and tracking purposes. The specimens are currently stored in a controlled environment at Yunnan Agricultural University laboratory.

**TABLE 1:** The source locations of 49 collected *D. chrysotoxum* flowers.

Site number	Longitude	Latitude	Soil type	Mean annual temperature	Precipitation	
	°E	°N		°C	mm	
1	99.49994	24.43325	Laterite	18	1,200	
2	99.44834	24.80358	Laterite	23	1,300	
3	103.4295	23.34617	Brown soil	21	1,000	
4	99.59503	22.33432	Loess	17	1,100	
5	101.2553	21.94129	Laterite	17.5	1,000	
6	100.05819	21.70253	Laterite	15.9	1,384	
7	104.70955	23.13159	Laterite	15.9	1,384	
8	100.09544	23.89047	Laterite	19	1,361	
9	99.25272	23.15288	Loess	22.3	1,100	
10	99.40945	23.54505	Loess	14.9	1,253	
11	102.50244	23.712	Laterite	16.9	1,463	
12	100.62879	23.74012	Laterite	14.5	1,302	
13	100.80644	22.017	Laterite	21	1,310	
14	103.16652	23.36494	Laterite	16.4	890	
15	99.24327	24.42862	Laterite	21.2	1,085	
16	107.25949	24.14732	Rice paddy soil	17.7	1,021	
17	107.54748	24.98185	Laterite	17	1,070	
18	99.17211	25.12765	Laterite	16.2	967	
19	104.60231	30.84263	Laterite	15.6	1,120	
20	104.40041	23.01899	Laterite	15.5	1,120	
21	109.43442	24.33196	Brown soil	14.5	2,100	
22	98.83216	24.70123	Laterite	15	2,000	
23	106.56092	28.60749	Laterite	16.9	1,900	
24	106.75855	25.43084	Laterite	16.5	1,444	
25	105.00849	25.51054	Chernozem	13.3	2,200	
26	103.49155	29.60698	Laterite	10.5	1,800	
27	99.04627	24.32563	Laterite	20.6	1,450	
28	100.17308	25.70164	Laterite	16.4	2,100	
29	98.76833	24.15595	Laterite	18	1,250	
30	98.45436	25.02419	Laterite	15.2	1,520	
31	100.59228	22.50461	Laterite	17.8	1,500	
32	100.84872	24.44269	Brown soil	18.3	1,087	
33	108.37345	22.82261	Laterite	19.7	1,363	
34	99.9385	22.56194	Laterite	19	1,400	
35	101.05215	23.05462	Laterite	12	1,414	
36	100.12953	24.44281	Laterite	16	1,367	
37	117.60103	28.93683	Laterite	17.4	1,283	
38	98.83187	23.76832	Laterite	18.9	1,626	
39	117.94946	28.46063	Laterite	19.5	2,050	
40	99.9317	24.58939	Laterite	16.5	1,307	
41	98.41255	24.39881	Laterite	19.6	1,655	
42	97.86249	24.02282	Laterite	20	1,453	
43	97.79853	24.18947	Laterite	18.9	1,595	
44	98.30313	24.81078	Laterite	18.3	1,437	
45	97.94994	24.69746	Laterite	19.3	1,464	
46	105.88798	28.52633	Purple soil	18.1	1,196	
47	103.57841	29.74385	Purple soil	17.1	1,375	
48	114.97096	25.82548	Laterite	19.8	1,319	
49	102.6155	24.79063	Laterite	15.1	1,100	

SPF‐grade SD rats, provided by Liaoning Changsheng Biotechnology Co. Ltd., with 10 males and 10 females (quality certificate No: 1107262011000885), production license No: SCXK (Liao) 2015‐0001, for acute oral toxicity test; SPF‐grade Kunming mice, provided by SBF (Beijing) Biotechnology Co. Ltd., with 55 females and 55 males (quality certificate No: 1103242011018470), 70 males (quality certificate No: 1103242011018471), production license No: SCXK (Jing) 2019‐0010, for mammalian erythrocyte micronucleus test and mouse spermatogonial cell chromosome aberration test; SPF‐grade SD rats, provided by SBF (Beijing) Biotechnology Co. Ltd., with 53 males and 53 females (quality certificate No: 110324200102502642), production license No: SCXK (Jing) 2019‐0010, for a 90‐day oral toxicity test. All animals were acclimatized for 3–5 days.

The experimental animal facility operates within a barrier system with the license number SYXK (Shaanxi) 2016‐007, maintaining a temperature of 20°C–26°C and relative humidity between 40% and 70%. The animal maintenance feed is supplied by Jiangsu Synergy Pharmaceutical Biotechnology Co. Ltd., with production license number Su Feed (2019) 01008, and feed quality certificates numbered 120200528004, 120200707005, 120200927016, and 120201024038. The experimental animal corncob bedding is provided by Dezhou Gumei Agricultural Technology Co. Ltd., with production batch numbers GMCC201903257‐2, GMCC202007693, and GMCC202008704.

All reagents and chemicals used for extractions were of commercial grade. Those for in vitro and cell culture assays were of analytical grade unless otherwise specified. Standards and solvents for UPLC analysis were of HPLC grade. TA98, TA100, TA102, TA1535, and TA1537 strains were purchased from Shanghai Baolu Biotechnology Co. Ltd. (Shanghai, China). Rat Liver Microsomal Enzyme (S9) was purchased from CHI Scientific Biotechnology Co. Ltd. 2‐Aminoanthracene (batch number 10197304) and Cyclophosphamide (CP) (batch number 5003M28N) were purchased from Alfa Aesar Chemical Co. Ltd. Dixon (batch number 4030165‐01) was purchased from Accustandard Biotechnology Co. Ltd. Sodium azide (batch number 20160720) was purchased from Tianjin Fengchuan Chemical Reagent Technology Co. Ltd. 2‐Aminofluorene (batch number 929A031) was purchased from Beijing Solarbio Science and Technology Co. Ltd. 9‐Aminoacridine (batch number BCBT2829) was purchased from Sigma‐Aldrich Co. Ltd. 1,8‐Dihydroxyanthraquinone (batch number C1523115) was purchased from Kuer Bioengineering Co. Ltd. Other chemicals such as paraffin, colchicine, Giemsa stain, 2,4,6‐trinitrophenol, glycerin, and alizarin red were purchased from Xi'an United Nations Quality Detection Technology Co. Ltd. (Xi'an, China).

Atomic fluorescence photometer (AFS‐933), Atomic absorption photometer (ZEEnit650P), Inductively‐Coupled Plasma Mass Spectrometer (CPMS‐2030), Biochemical incubator (SPX‐250B‐Z), Automatic biochemical analyzer (BS‐330E), Automatic hematology analyzer (ADVIA2120i), Urine Analyzer (URIT‐500B), Automatic coagulation analyzer (COMPACT MAX), Tissue automatic dehydrator (Histo Core PEARL). Aspartic acid (Asp), threonine (Thr), serine (Ser), glutamic acid (Glu), proline (Pro), glycine (Gly), alanine (Ala), valine (Val), methionine (Met), isoleucine (ILe), leucine (Leu), tyrosine (Tyrosine, Tyr) The standard products of Phenylalanine (Phe), Histidine (His), Lysine (Lys), and Arginine (Arg) are purchased from Agilent in the United States. ABTS cationic radical scavenging assay kit (batch number: G0127W), DPPH radical scavenging assay kit (batch number: G0128W), superoxide anion scavenging assay kit (batch number: G0116W), and hydroxyl radical scavenging assay kit (batch number: G0125W) were purchased from Suzhou Grace Biotechnology Co. Ltd. All other reagents were provided by United Nations Quality Detection Technology Co. Ltd. (Xi'an, China), including Folin, methanol, sodium carbonate, photographic acid, ninhydrin, anthrone, ferrous target, sulfonic acid, paraffin, colchicine, giemsa stain, 2,4,6‐trinitrophenol, glycerin, and alizarin red.

Atomic fluorescence photometer (AFS‐933), Atomic absorption photometer (ZEEnit650P), inductively coupled plasma mass spectrometer (CPMS‐2030), Biochemical incubator (SPX‐250B‐Z), Automatic biochemical analyzer (BS‐330E), Automatic hematology analyzer (ADVIA2120i), Urine Analyzer (URIT‐500B), Automatic coagulation analyzer (COMPACT MAX), Tissue automatic dehydrator (Histo Core PEARL), UV‐visible spectrophotometer (756CRT), high‐speed liquid chromatography system (Tedia Company Inc., USA), Electronic analytical balance (A13204‐N); Enzyme reader (MD190), low‐temperature centrifuge (TLG‐16); Electric hot air drying oven (DHG‐9140); Vacuum dryer (YZG‐FZG); Vacuum freeze‐drying machine (FD‐1A‐50), Thermo Scientific Microplate Reader (Multiskan GO).

### 2.2 Safety Evaluation

The 49 fresh *D. chrysotoxum* flowers were subjected to a 60°C hot air drying treatment, and the content of prohibited pesticides and heavy metals was detected.

#### 2.2.1 Prohibited Pesticides

According to the method “Determination of Residual Amounts of Forbidden Pesticides in Chinese Materia Medica and Decoction Pieces (Plant Type)” of the 2020 edition of the Chinese Pharmacopoeia (2,341), the content of 33 prohibited pesticides in the samples is determined (Wang et al. 2021).

#### 2.2.2 Heavy Metals

The measurement of heavy metal residues, such as Cd, As, Hg, Cu, and Pb, was conducted using an Inductively Coupled Plasma Mass Spectrometer following the method outlined by Yuan et al. (2023).

### 2.3 Toxicological Research

Randomly select one of 49 *D. chrysotoxum* flowers for toxicological evaluation, chemical composition analysis, and antioxidant activity determination. Referring to the recommended oral dose of *D. officinale* , set at 3.0 g day^−1^, calculated based on an adult weight of 60 kg, the equivalent dose is 0.05 g kg^−1^ BW^−1^ day^−1^. For sample preparation, 1500 g of *D. chrysotoxum* flowers are soaked in 15 L of pure water at atmospheric pressure and a temperature of 80°C–90°C for 30 min, extracted twice, and the extract liquids are combined and concentrated to 1 L for further use. Each 1 mL of the concentrated extract is equivalent to 1.5 g of the original sample. The dose level used in the toxicological evaluation was determined based on the standards established by John Timbrell in “Introduction to Toxicology” (Timbrell and Barile 2023).

#### 2.3.1 Acute Oral Toxicity Test

After the adaptation period, 20 SD rats, half male and half female, with a post‐fast weight of 180–220 g, are selected. Dosage design: The concentrated extract is orally administered to the animals at a dosage of 20 mL kg^−1^ BW^−1^ (equivalent to 30.0 g kg^−1^ BW^−1^ of *D. chrysotoxum* flowers). Following oral administration, the animals are observed for signs of toxicity and mortality for 14 consecutive days. The body weights of the rats are measured at the beginning of the experiment, on the 7th day, and on the 14th day. At the end of the experiment, the animals are euthanized for gross dissection.

#### 2.3.2 Bacterial Reverse Mutation Test

Using the authenticated strains TA98, TA100, TA102, TA1535, and TA1537, the standard plate incorporation assay is conducted with and without rat liver S9 mix (at a volume of 0.5 mL dish^−1^) for two experiments. The dosage design for the first experiment includes five dose groups: 5000, 1581, 500, 158, and 50 μL dish^−1^, along with sterile distilled water used as the solvent control group (SC), spontaneous revertant group (SR), and positive control group (PC). For the second experiment, the doses are 5000, 1000, 200, 40, and 8 μL dish^−1^, along with the solvent (sterile distilled water) control group, spontaneous revertant group, and positive control group.

Sample preparation: 1 mL of concentrated extract is diluted with distilled water to 20 mL to obtain a 5% solution. For the first experiment, subsequent dilutions are made by $\sqrt{10}$ fold dilution starting from the 5% solution. For the second experiment, subsequent dilutions are made by a fivefold dilution starting from the 5% solution. All prepared test samples undergo autoclaving at high pressure (0.103 MPa, 20 min).

During the experiment, 0.1 mL of each concentration solution is added to the plates as different doses of the samples, with each dose tested in triplicate plates. For the assays without S9 mix, the positive controls are 1.5 μg dish^−1^ of sodium azide (for TA100 and TA1535), 50.0 μg dish^−1^ of dexamethasone (for TA98 and TA102), and 50.0 μg dish^−1^ of 9‐aminoacridine (for TA1537). For the assays with S9 mix, the positive controls are 20.0 μg dish^−1^ of 2‐aminofluorene (for TA98 and TA100), 50.0 μg dish^−1^ of 1,8‐dihydroxyanthraquinone (for TA102), and 2.0 μg dish^−1^ of 2‐aminofluorene (for TA1535 and TA1537). The volume of positive controls added to each dish is 0.1 mL.

#### 2.3.3 Mammalian Erythrocyte Micronucleus Test

Fifty Kunming mice, weighing 25–35 g, half male and half female, were selected. The mice were randomly divided into 5 groups, each consisting of 10, with an equal number of males and females. Dose design included three dosage groups: 6.67 mL kg^−1^ BW^−1^, 3.33 mL kg^−1^ BW^−1^, and 1.67 mL kg^−1^ BW^−1^, along with a solvent (pure water) control group and a cyclophosphamide positive control group (CP, 40 mg kg^−1^ BW^−1^). Sample preparation involved measuring 6.67 mL, 3.33 mL, and 1.67 mL of concentrate, adding pure water to 20 mL, and mixing well to create solutions with concentrations of 33.35%, 16.65%, and 8.35%, respectively, and 0.04 g of CP was dissolved in 20 mL of pure water. Gavage volume was set at 20 mL kg^−1^ BW^−1^, and the test was conducted using a 30‐h gavage method, with a 24‐h interval between the two administrations. Animals were euthanized by cervical dislocation 6 h after the second dose of the test sample, and femoral bone marrow smears were taken, fixed, and Giemsa stained. Under oil immersion, the number of micronucleated cells among 2,000 polychromatic erythrocytes (PCE) was counted for each mouse to calculate the micronucleus frequency per thousand. Observation of 200 erythrocytes was done to count the proportion of PCEs in total erythrocytes, that is, the PCE/RBC ratio. NCE stands for normochromatic erythrocytes. The number of rats tested is 5, and the number of PCEs tested is 2,000 × 5 in each group.

#### 2.3.4 Mouse Spermatogonial Chromosome Aberration Test

Thirty male Kunming mice, weighing 25–35 g, were selected and randomly divided into 5 groups, with the high‐dose group containing 10 and the remaining groups containing 5 each. The dosage of the flower extract and the control group and the preparation method were consistent with the mammalian erythrocyte micronucleus test. The gavage volume was set at 20 mL kg^−1^ BW^−1^, and the test sample was administered orally in a single gavage. Additionally, 0.04 g of CP was taken, dissolved in 10 mL of 0.9% sodium chloride injection to prepare a 0.4% solution, and administered as a single intraperitoneal injection at 10 mL kg^−1^ BW^−1^. Animals in the high‐dose group were euthanized for sampling at 24 h and 48 h after the last administration of the flower extract, with 5 animals at each sampling time point. Animals in the other groups were euthanized for sampling 24 h after the last administration of the flower extract. All animals were injected intraperitoneally with colchicine solution (dose: 5 mg kg^−1^ BW^−1^, injection volume: 10 mL kg^−1^ BW^−1^) 5 h before euthanasia. Animals were euthanized by cervical dislocation, and the testes were removed, placed in 1% sodium citrate solution for membrane removal, seminiferous tubules separation, and hypotonic treatment in a 37°C water bath for 30 min. After carefully removing the hypotonic solution and undergoing fix‐centrifuge‐fix‐centrifuge‐fix cycles, the supernatant was discarded, leaving 0.5–1.0 mL of cell suspension to drop on cold slides. After drying, the slides were Giemsa stained. Under oil immersion, 100 metaphase cells were counted for each animal to record chromosome aberration types, numbers, and aberrant cell rates. 1,000 cells per animal were observed to determine the spermatogonial mitotic index, which in the high‐dose group should not be less than 50% of the control group. The number of rats tested is 5, the number of observed cells is 1000 × 5, and the number of metamyelocytes detected during examination is 100 × 5 in each group.

#### 2.3.5 90‐Day Oral Toxicity Test

One hundred weaned SD rats, half male and half female, weighing 61–83 g, were selected and randomly divided into four main test groups and two mid‐term observation groups. Each main test group had 20 animals, half male and half female, and each mid‐term observation group had 10 animals, half male and half female. Test design: The test groups used doses of 10.00 mL kg^−1^ BW^−1^ (undiluted sample concentrate), 6.67 mL kg^−1^ BW^−1^, and 3.33 mL kg^−1^ BW^−1^, with an additional solvent (pure water) control group; mid‐term observation groups were set at a 10.00 mL kg^−1^ BW^−1^ dose and a solvent (pure water) control group, with a gavage volume of 10 mL kg^−1^ BW^−1^. Sample preparation: The undiluted concentrate was used for the high dose, and 66.7 and 33.3 mL of the concentrate were each diluted with pure water to 100 mL to make solutions of 66.7% and 33.3% concentrations, respectively. Gavage was performed 6 days a week; the main test groups were maintained for 90 days, during which daily observations of the animals’ general appearance, behavior, symptoms of poisoning, and mortality were made. Body weight was measured twice a week for the first 4 weeks and once a week thereafter. Weekly food intake and leftover amounts were measured to calculate weekly and total food efficiency rates. Ophthalmological examinations were conducted on the high‐dose and solvent control groups of the main test and mid‐term observation groups before and after the test. Midway through the test (Day 43), hematological, biochemical, and urine indicators were measured for the mid‐term observation group animals. After fasting for 16 h at the end of the test, fasting weights were recorded. Blood was drawn from the abdominal aorta under anesthesia for hematological parameters using an automatic blood analyzer, coagulation markers prothrombin time and activated partial thromboplastin time using an automatic coagulometer, and biochemical parameters aspartate aminotransferase, alanine aminotransferase, alkaline phosphatase, gamma‐glutamyltransferase, serum urea, total protein, albumin, triglycerides, creatinine, total cholesterol, glucose, potassium, sodium, chloride using an automatic biochemical analyzer. Urine indicators were measured using a urine analyzer. Animals were dissected to observe internal organ changes, and the weights of the liver, kidneys, spleen, adrenal glands, brain, heart, thymus, uterus, ovaries, testes, and epididymis were recorded, calculating their organ/body weight ratios. Tissues from the brain, pituitary, thyroid, thymus, lungs, heart, liver, spleen, kidneys, adrenal glands, stomach, duodenum, jejunum, ileum, colon, rectum, pancreas, mesenteric lymph nodes, ovaries, uterus, testes, epididymis, prostate, and bladder were collected for histopathological examination. Animals had free access to food and water throughout the test period.

### 2.4 Widely Targeted Metabolomic Test

The preparation method for the mixed sample extract is: 1 g of *D. chrysotoxum* flowers was extracted with 100 mL of distilled water at 80°C for 2 h and filtered through filter paper. The analysis of chemical compounds and the evaluation of antioxidant activity utilized the same sample extracting method. Metabolites in the *D. chrysotoxum* flowers were detected and analyzed using a widely targeted metabolomics approach, conducted via UPLC‐MS/MS by Maiwei Metabolic Biotechnology Co. Ltd. (www.metware.cn/, Wuhan, China). Sample extraction, UPLC conditions, electron spray ionization (ESI)‐triple quadrupole‐linear ion trap mass spectrometry methods, and metabolite data analysis adhered to their standard procedures as previously detailed by Cao et al. (2019). A quality control (QC) analysis was performed to ensure data reliability. The QC sample, created by blending extracts, was intermittently introduced every 10 samples to monitor variations in repeated analyses.

### 2.5 Analysis of Chemical Compounds

The quantification of polysaccharides in *D. chrysotoxum* flowers follows the guidance of (He et al. 2022). The phenol‐sulfuric acid reaction was performed: 5% phenol solution (1.0 mL) and concentrated sulfuric acid (5.0 mL) were added sequentially to the *D. chrysotoxum* flowers extract (1.0 mL), rapidly shaken, heated in boiling water for 20 min, immediately removed, and ice bathed for 5 min. The absorbance of the reaction solution was measured at 488 nm. The soluble sugar content was detected by the anthrone colorimetric method (Zhou et al. 2023). In this method, 1 mL extracting solution was mixed with 1 mL distilled water, 0.5 mL anthrone ethyl acetate, and 5 mL concentrated sulfuric acid, and the soluble sugar content was calculated by absorbance at 630 nm using the Thermo Scientific Microplate Reader (Multiskan GO). Total polyphenol content of samples was determined using the Folin–Ciocalteu method (Man et al. 2016). Briefly, the extracting solution (50 μL) was mixed with 850 μL of dd H_2_O and 50 μL of Folin–Ciocalteu reagent. Subsequently, 150 μL of 10% Na_2_CO_3_ solution was added to the mixture and placed in the dark at ambient temperature for 20 min. The absorbance of the reaction mixture was read at 560 nm against deionized water as a blank. The content of carotenoids in the *D. chrysotoxum* flowers extract was determined by high‐performance liquid chromatography (HPLC) system following the method of Kan et al. (2018). The total alkaloid content of *D. chrysotoxum* flowers was determined using the acid dye colorimetric method described previously by Tian et al. (2018). Briefly, 1.0 mL of the extract solution (200 μg mL^−1^) was mixed with 5 mL of a NaOAc/AcOH buffer solution (pH 3.5) and 5 mL bromocresol green indicator. The mixture was shaken for 3 min, then 5 mL of Trichloromethane (CHCl_3_) was added and shaken for 2 min after mixing. The mixture was maintained for 30 min before removing the CHCl_3_ layer, then the absorbance of the mixture at 420 nm was determined with a UV spectrophotometer. The total alkaloid content of *D. chrysotoxum* flowers was determined using the acid dye colorimetric method described previously by Rao et al. (2022). Briefly, each 0.5 mL extract solution was mixed with 0.15 mL 5% sodium nitrite solution for 6 min. They were mixed with 0.15 mL 10% aluminum nitrate solution for 6 min. They were further mixed with 2 mL 4% sodium hydroxide solution and 2.2 mL distilled water for 3 min. The absorbance was determined at 508 nm. The amounts of amino acids in *D. chrysotoxum* flowers were measured using a high‐performance liquid chromatography with fluorescence detection (HPLC–FLD) method with online o‐phthalaldehyde (OPA) derivatization in accordance with our previous work (Zhao et al. 2021).

### 2.6 Evaluation of Antioxidant Activity

Using commercial assay kits provided by Suzhou Grace Bio‐tech Co. Ltd. (Suzhou, China), the clearance capacities of hydroxyl free radicals, superoxide anions, ABTS free radicals, and DPPH free radicals in the *D. chrysotoxum* flower extract were determined according to the manufacturer's instructions (Zhao et al. 2021).

### 2.7 Statistical Analysis

The mammalian erythrocyte micronucleus test employed a χ^2^ test, while the mouse spermatogonial chromosomal aberration test utilized a rank sum test. Data from the 90‐day oral toxicity test underwent homogeneity of variance testing; if the variance was homogeneous, ANOVA was conducted, and if the P‐value was less than 0.05, Dunnett's method was used for pairwise comparisons. If the variance was not homogeneous, data transformation was performed; if homogeneity was still not achieved, independent samples t‐test or rank sum test was employed. If the P‐value was less than 0.05, Dunnett's T3 method was used for pairwise comparisons. All statistical analyses were conducted using SPSS 26 software.

## 3 Results

### 3.1 Safety Evaluation

**FIGURE 1:**
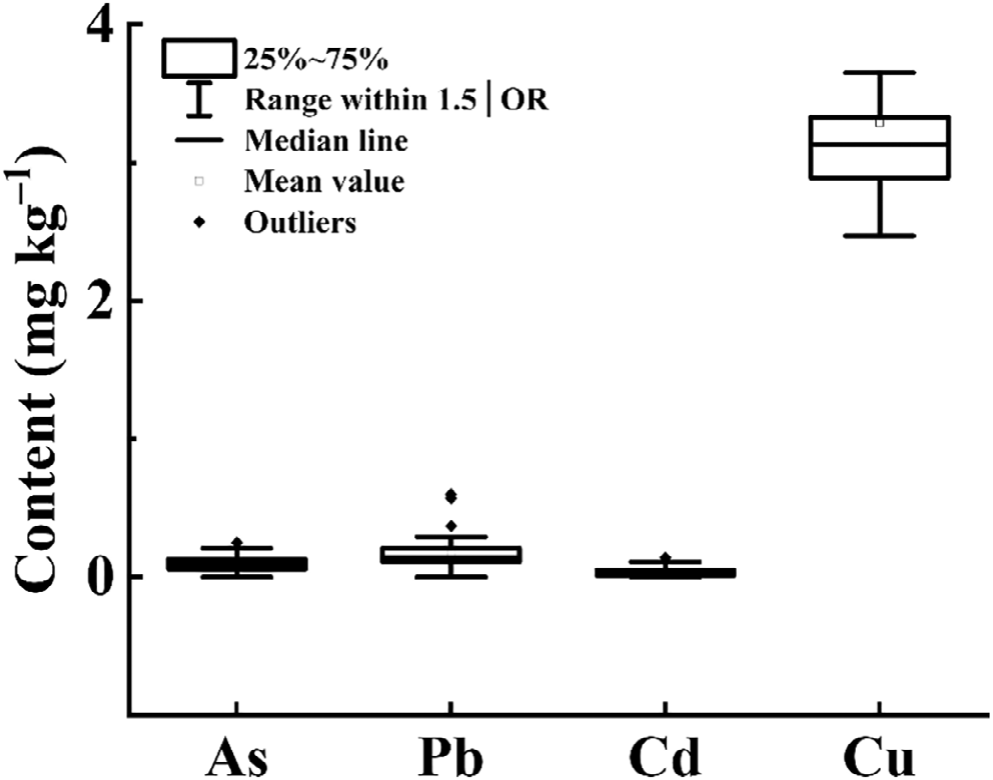
The contents of As, Pb, Cd, and Cu in the 49 samples of *D. chrysotoxum* flowers.

Among the 49 samples of *D. chrysotoxum* flowers, the content of arsenic ranges from 0 to 0.26 mg kg^−1^, Pb ranges from 0 to 0.77 mg kg^−1^, Hg was not detected, Cd ranges from 0 to 0.14 mg kg^−1^, and Cu ranges from 2.47 to 12.40 mg kg^−1^ (Figure 1). The levels of these five heavy metals are all within permissible limits. Additionally, none of the 49 samples of *D. chrysotoxum* flowers showed detectable residues of the 33 banned pesticides (Undetected data not displayed). Overall, there were no detectable pesticide residue levels or residues above safety thresholds in any of the tested samples.

### 3.2 Toxicological Research

#### 3.2.1 Acute Oral Toxicity Test

After administering the *D. chrysotoxum* flower extract orally to SD rats at a dose of 30.0 g kg^−1^ BW^−1^ (equivalent to 600 times the recommended human dose), the animals exhibited good growth, with no apparent impact on body weight. There were no observable signs of toxicity, and over the 14‐day observation period, there were no fatalities among the animals (Table S1). After the experiment, upon dissecting the animals, gross examination of major organs such as the liver, kidneys, spleen, lungs, and intestines revealed no significant abnormal changes (Figure S1).

#### 3.2.2 Bacterial Reverse Mutation Test

**FIGURE 2:**
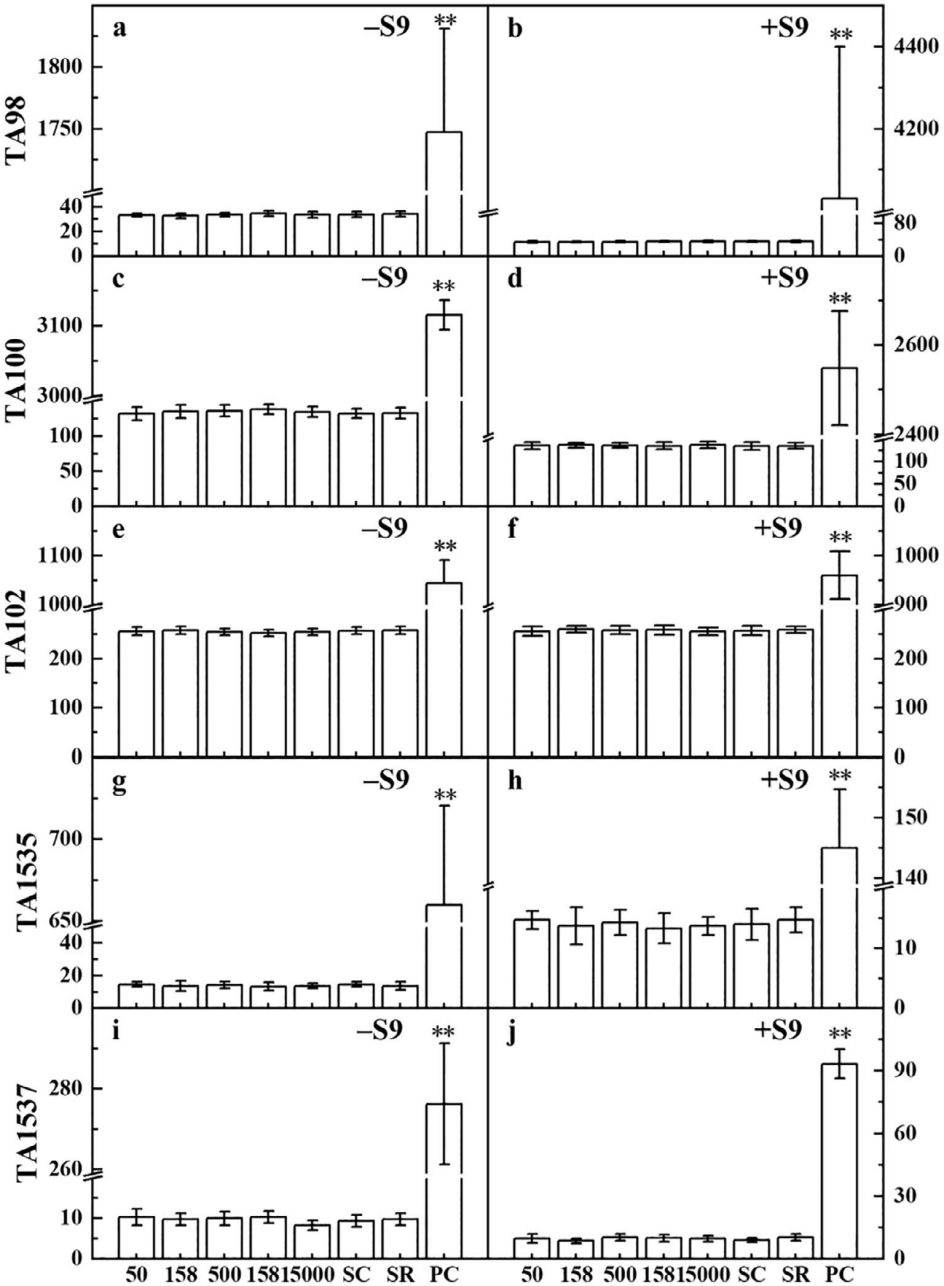
Number of revertant colonies per dish in the bacterial reverse mutation test of *D. chrysotoxum* flower extract. The results of both bacterial reverse mutation test experiments were negative, so only the results of one experiment were displayed. The presence of "**" on the column in the bar chart indicates significant differences between this treatment and other treatments.

Regardless of the presence or absence of S9 activation, the revertant colony counts in all dose groups did not exceed twice the spontaneous revertant colony count (Figure 2). Furthermore, there was no dose–response relationship observed. These findings indicate that the mutagenic test results of the *D. chrysotoxum* flowers extract were negative.

#### 3.2.3 Mammalian Erythrocyte Micronucleus Test

Comparing the micronucleus frequencies of bone marrow cells in mice across all dosage groups with the solvent control group, there were no significant differences (Table 2). The micronucleus frequency in the cyclophosphamide positive control group was significantly different from that in the solvent control group. The PCE/RBC values in all dosage groups were within the normal range, and the proportion of PCEs in the total erythrocytes was not less than 20% of that in the solvent control group, with no significant difference observed. These results indicate that the sample tested negative in the mammalian erythrocyte micronucleus test.

**TABLE 2:** Mammalian erythrocyte micronucleus test results of *D. chrysotoxum* flower extract.

Gender	Dose (g kg^−1^ BW^−1^)	Micronucleus count	Micronucleus rate (%)	PCE	NCE	PCE/RBC
Female	6.67	10	1.00 ± 0.35	103.8 ± 6.4	96.2 ± 6.4	0.52 ± 0.03
	3.33	9	0.90 ± 0.42	99.6 ± 6.9	100.4 ± 6.9	0.50 ± 0.03
	1.67	11	1.10 ± 0.42	100.4 ± 5.9	99.6 ± 5.9	0.50 ± 0.03
	0	11	1.10 ± 0.22	104.4 ± 5.8	95.6 ± 5.8	0.52 ± 0.03
	CP	257**	25.70 ± 2.08**	97.8 ± 5.9	102.2 ± 5.9	0.49 ± 0.03
Male	6.67	10	1.00 ± 0.35	101.6 ± 5.9	98.4 ± 5.9	0.51 ± 0.03
	3.33	12	1.20 ± 0.27	105.6 ± 4.7	94.4 ± 4.7	0.53 ± 0.02
	1.67	9	0.90 ± 0.42	98.4 ± 5.1	101.6 ± 5.1	0.49 ± 0.03
	0	10	1.00 ± 0.35	103.0 ± 5.8	97.0 ± 5.8	0.52 ± 0.03
	CP	252**	25.20 ± 1.68**	95.4 ± 5.3	104.6 ± 5.3	0.48 ± 0.03

*Note:* The presence of “**” after the data indicates significant differences between this treatment and other treatments.

#### 3.2.4 Mouse Spermatogonial Chromosome Aberration Test

The mitotic index of spermatogonial cells in the high‐dose group did not fall below 50% of the solvent control (Table 3). Comparisons of the aberrant cell rates and chromosome aberration rates across all dosage groups with the solvent control group showed no significant differences, while the cyclophosphamide positive control group was significantly higher than the solvent control group. The main types of chromosome aberrations observed were breaks and fragments (Table 2). These results indicate that the sample tested negative in the mouse spermatogonial chromosome aberration test.

**TABLE 3:** Mouse spermatogonial chromosome aberration test results of *D. chrysotoxum* flower extract.

Dose	Mitotic index	Abnormal cell count	Abnormal cell rate	Chromosomal aberrations							Total aberrations	Chromosomal aberration rate
g kg^−1^ BW^−1^	%		%	Breaks	Fragments	Rings	Multiple centromeres	Monad interchange	Acentric ring	Micronuclei		(%)
6.67 (24 h)	31.4 ± 0.9	1	0.2 ± 0.5	1	0	0	0	0	0	0	1	0.2 ± 0.5
6.67 (48 h)	30.8 ± 1.2	2	0.4 ± 0.6	1	1	0	0	0	0	0	2	0.4 ± 0.6
3.33	33.1 ± 1.8	1	0.2 ± 0.5	1	0	0	0	0	0	0	1	0.2 ± 0.5
1.67	32.6 ± 1.6	2	0.4 ± 0.6	1	0	0	0	0	0	1	2	0.4 ± 0.6
0	31.8 ± 1.3	2	0.4 ± 0.6	1	1	0	0	0	0	0	2	0.4 ± 0.6
CP (0.04 g)	32.1 ± 1.0	43**	8.6 ± 1.1**	21**	17**	0	0	0	0	17**	55**	11.0 ± 1.6**

*Note:* The presence of “**” after the data indicates significant differences between this treatment and other treatments.

#### 3.2.5 90‐Day Oral Toxicity Test

**FIGURE 3:**
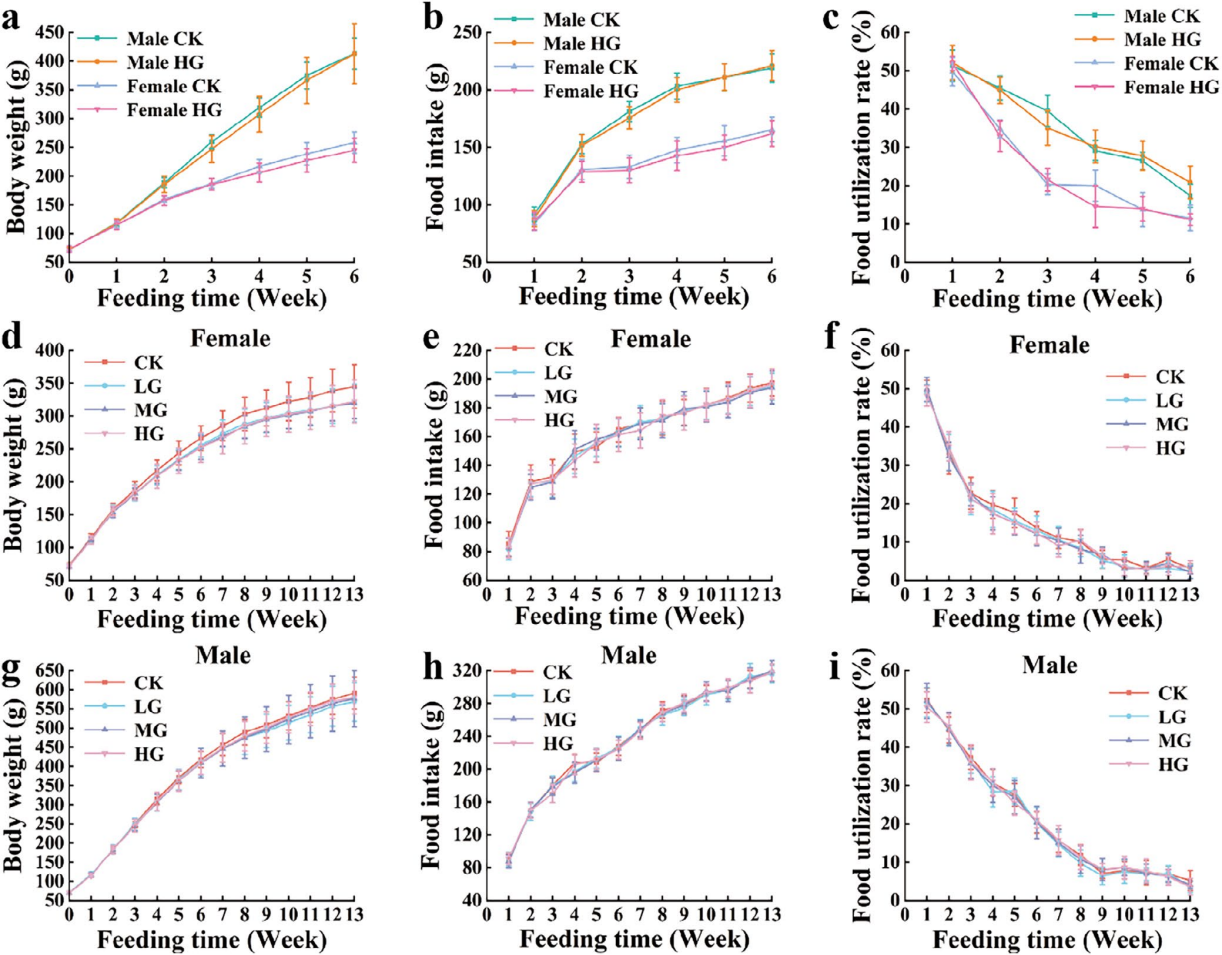
The body weight changes and food utilization rates of rats in the 90‐day oral toxicity test of *D. chrysotoxum* flowers extract. CK, LG, MG, HG represent 0, 3.33, 6.67, 10 g kg^−1^ BW^−1^, respectively.

During the 90‐day oral toxicity test, the animals remained in good health, with continuous weight gain, and no signs of toxicity were observed after daily oral administration of different doses of the *D. chrysotoxum* flowers extract in each dosage group (Figure 3). Additionally, we have observed that ophthalmological examinations of the high‐dose and solvent control groups in the main test and mid‐term observation groups revealed no abnormal changes in the cornea, lens, conjunctiva, or iris.

**FIGURE 4:**
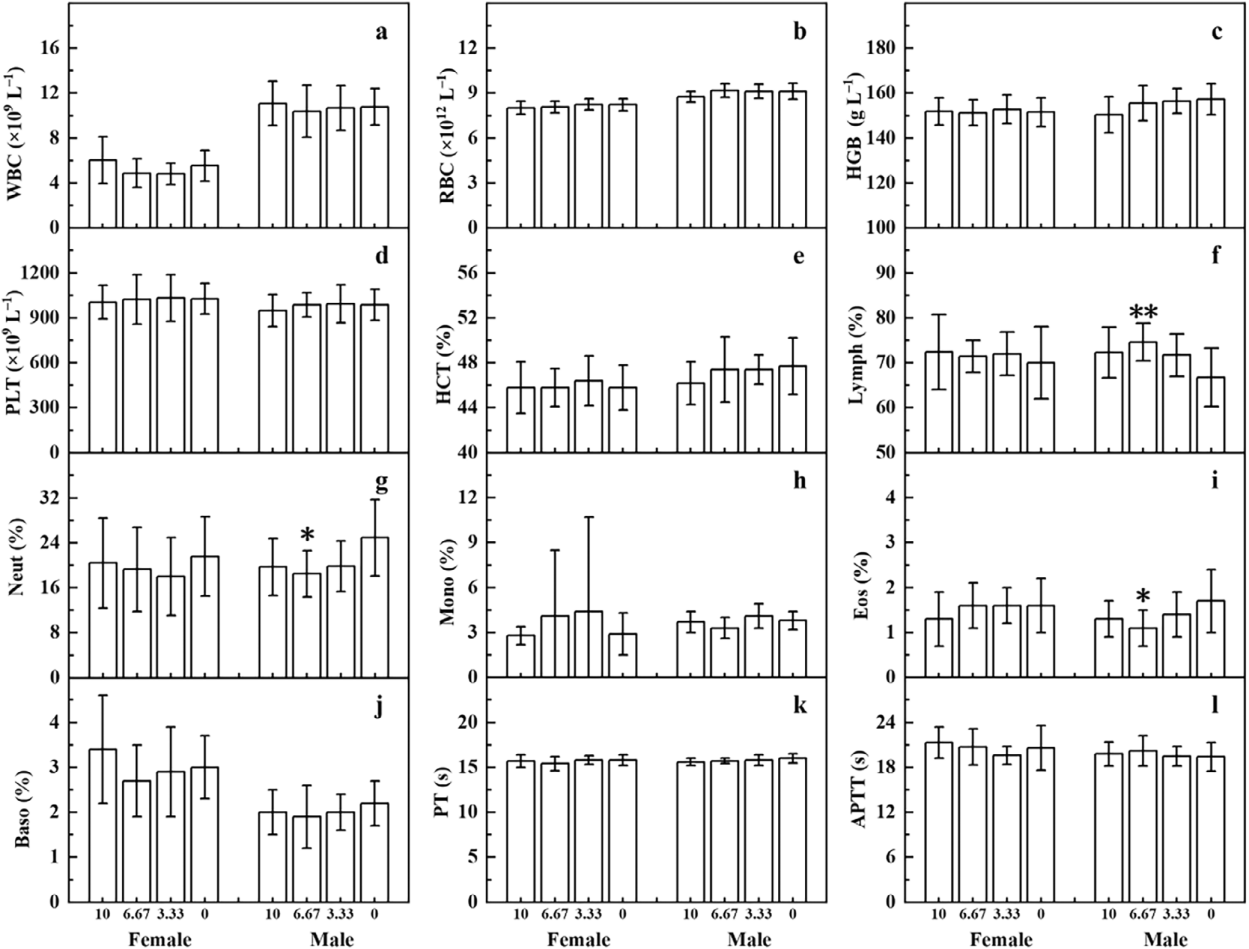
The hematological parameters of rats in the 90‐day oral toxicity test of *D. chrysotoxum* flower extract in main test groups. WBC stands for white blood cells, RBC stands for red blood cells, HGB stands for hemoglobin, PLT stands for platelets, HCT stands for hematocrit, Lymph stands for lymphocytes, Neut stands for neutrophils, Mono stands for monocytes, Eos stands for eosinophils, Baso stands for basophils, PT stands for prothrombin time, and APTT stands for activated partial thromboplastin time.

**FIGURE 5:**
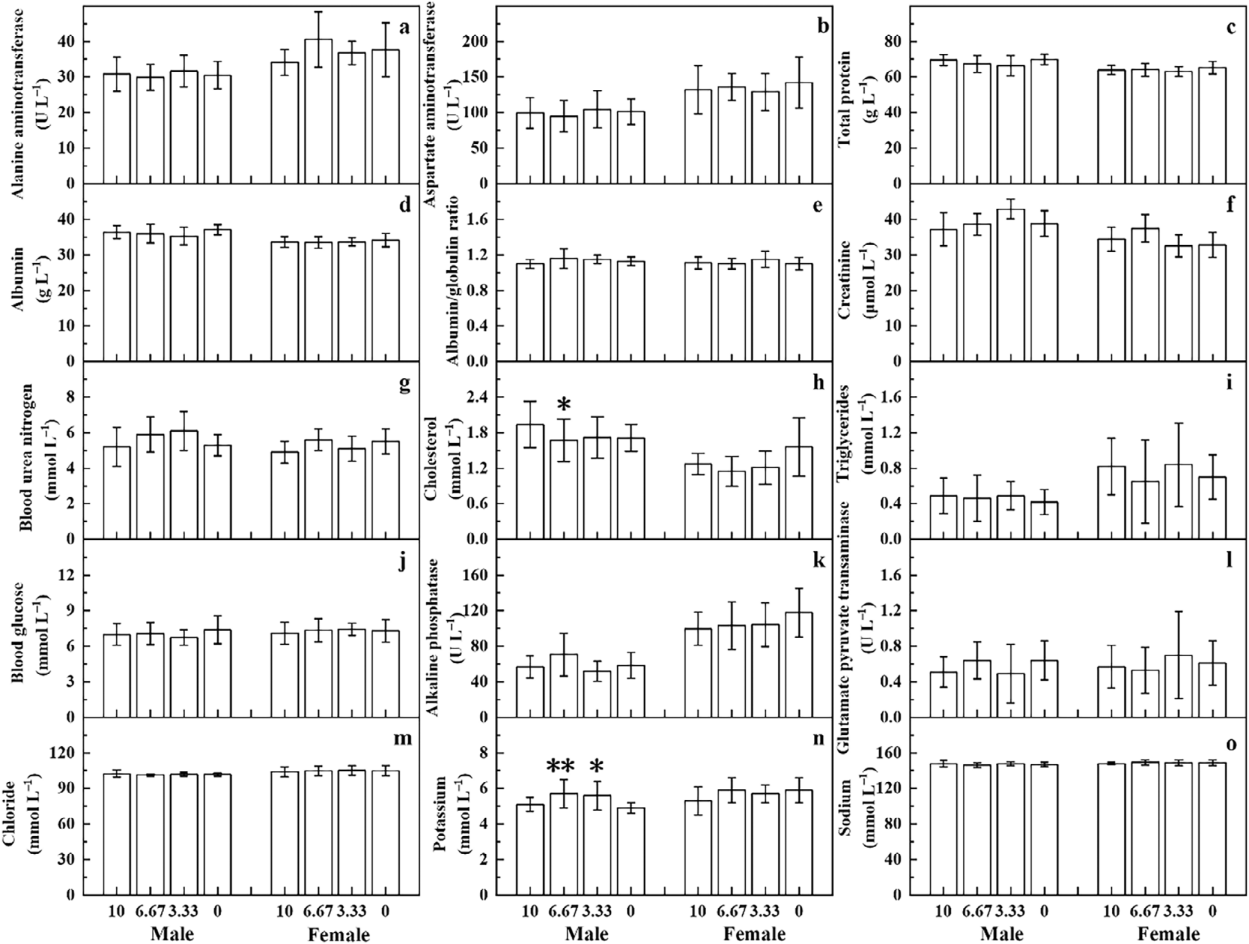
The blood biochemical parameters of rats in the 90‐day oral toxicity test of *D. chrysotoxum* flower extract in main test groups.

Comparing with the solvent control group, there were no significant differences in hematological, biochemical parameters, and urine parameters in the high‐dose group of male and female rats in the mid‐term observation group (Figures S2, S3, and Table S2). In the main test group, significant differences were observed in some hematological, biochemical parameters, and urine parameters in certain dosage groups of male and female rats, but these differences were within the background data range of this laboratory and had no biological significance (Figures 4, 5, and Table S2). Other parameters showed no significant differences.

The organ wet weights and organ/body weight ratios of male and female rats in each dosage group of the main test group showed no significant differences compared to the solvent control group (Tables S3 and S4). After euthanizing the animals at the end of the experiment and performing gross dissections, no abnormal changes were observed macroscopically. Therefore, histopathological examinations were only conducted on the brain, pituitary gland, thyroid gland, thymus, lungs, heart, liver, spleen, kidneys, adrenal glands, stomach, duodenum, jejunum, ileum, colon, rectum, pancreas, mesenteric lymph nodes, ovaries, uterus, testes, epididymis, prostate, and bladder of the high‐dose and solvent control groups. The histopathological examination results showed mild hepatocellular steatosis in 3 female and 5 male rats in the high‐dose group and 4 rats of each sex in the solvent control group. The pancreas exhibited significant vacuolar changes in 2 female and 2 male rats in the high‐dose group and 1 female and 2 male rats in the solvent control group. Pulmonary alveolar hyperplasia was observed in 5 rats of each sex in the high‐dose group and 6 female and 4 male rats in the solvent control group. Bladder sediment was observed in 2 male rats in the high‐dose group and 4 male rats in the solvent control group. These tissue lesions were considered spontaneous mild lesions in animals. No other significant pathological changes were observed in the remaining tissues.

In conclusion, no evidence of toxic pathological damage was observed in animals administered with the high dose of the sample.

### 3.3 Chemical Composition

**FIGURE 6:**
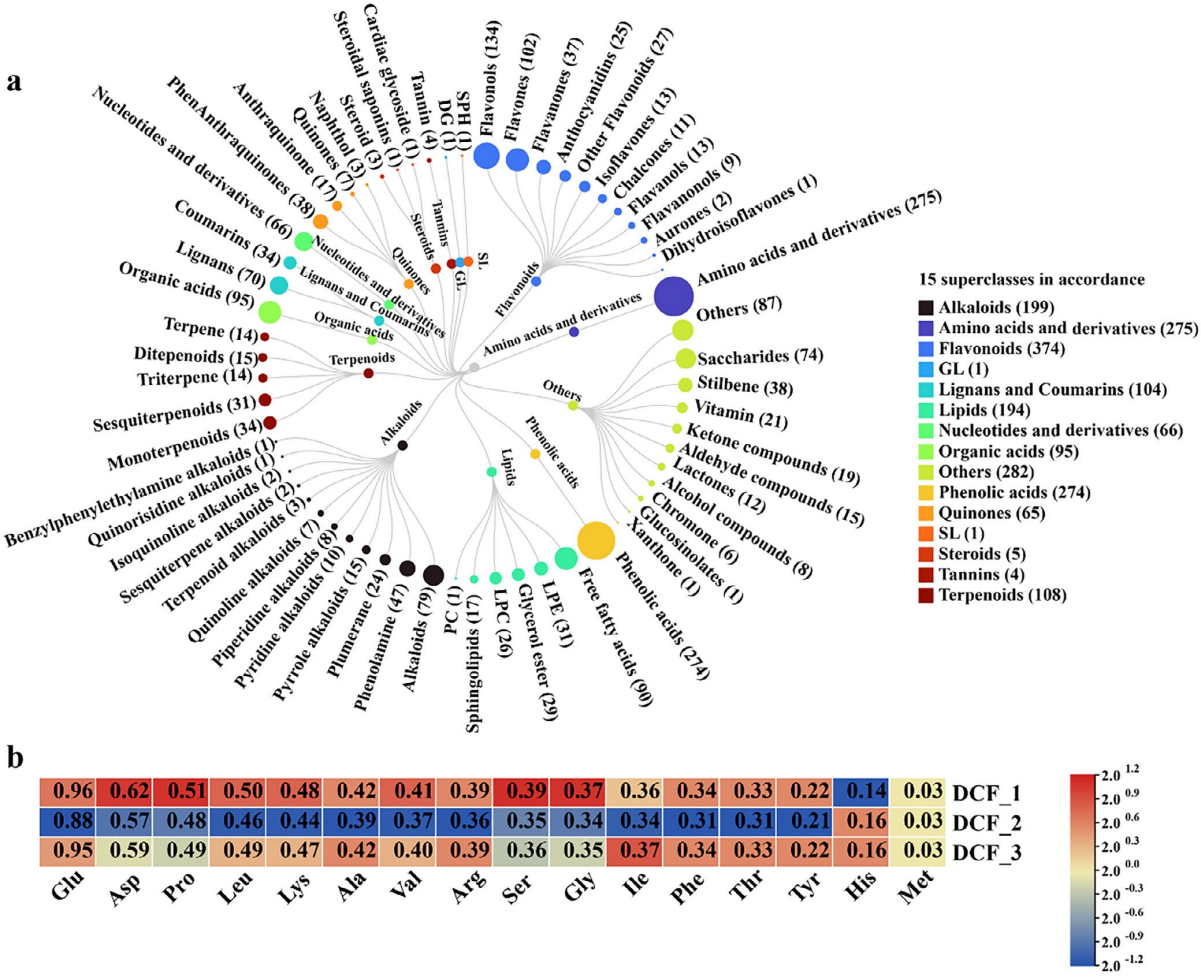
The widely targeted metabolomic test results (a) and amino acid content (b) of *D. chrysotoxum* flower extract.

Herein, 2047 metabolites were identified in *D. chr*ysotoxum flowers by the widely targeted metabolomics method (Figure 6a). These metabolites were categorized into 15 superclasses according to The Human Metabolome Database classification system. The major classes included 95 organic acids, 104 lignans and coumarins, 66 nucleotides and their derivatives, 275 amino acids and their derivatives, 1 glyceride, 199 alkaloids, 5 steroids, 194 lipids, 108 terpenes, 274 phenolic acids, 65 quinones, 1 sphingolipid, 4 tannins, 374 flavonoids, and 282 other substances.

We detected the content of six common compounds in *D. chrysotoxum* flowers, among which the polysaccharide content (%) was 25.25 ± 1.36, the soluble sugar content (%) was 52.09 ± 1.47, the polyphenol content (%) was 2.67 ± 0.07, the carotenoid content (%) was 0.06 ± 0.01, the total alkaloid content (mg kg^−1^) was 1.51 ± 0.02, and the total flavonoid content (mg g^−1^) was 4.50 ± 0.20. HPLC analysis revealed that the free amino acid contents in *D. chrysotoxum* flowers were 0.77% ± 0.01%. Sixteen amino acids were detected in *D. chrysotoxum* flowers (Figure 6b), with the highest contents observed for glutamate, at 0.93 mg g^−1^, and aspartate, at 0.60 mg g^−1^.

### 3.4 Antioxidant Activity Evaluation

**FIGURE 7:**
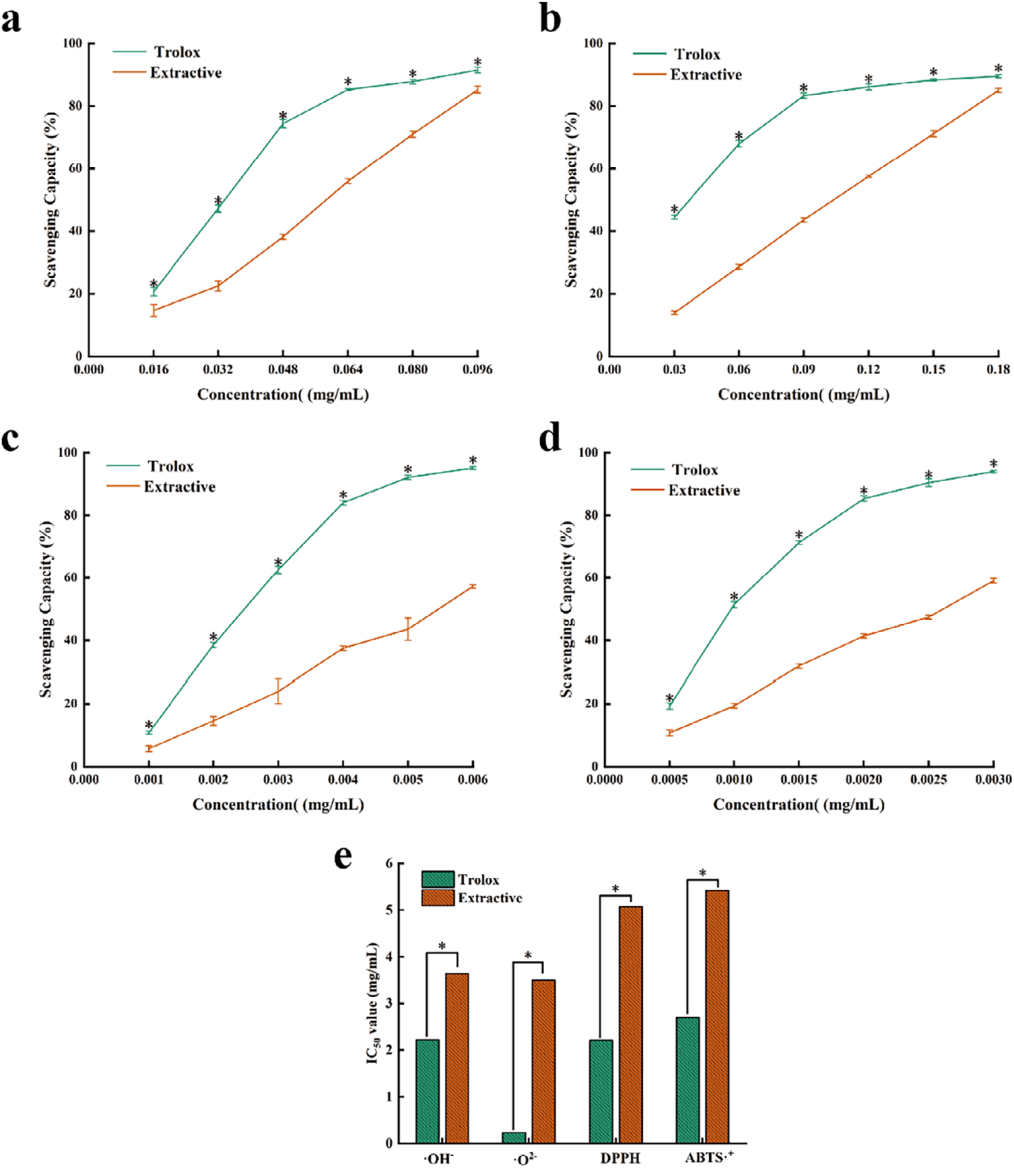
Scavenging activities of·OH^−^ radicals (a), ·O^2−^ radicals (b), DPPH radicals (c), ABTS^+^ radicals (d), and EC_50_ (e) in the *D. chrysotoxum* flower extract, with Trolox as the positive control. *Indicates a significant difference based on ANOVA post hoc test (including *t*‐test) at *p* < 0.05.

*D. chrysotoxum* flower extract is rich in nutrients and active components, including polysaccharides, polyphenol, amino acids, and multivitamins, etc. In vitro assays, *D. chrysotoxum* flower extract showed good scavenging activities (SAs) for hydroxyl radicals (Figure 7a), superoxide anion radicals (Figure 7b), DPPH (Figure 7c), and ABTS + (Figure 7d) with dose‐dependent behavior. The EC50 for SAs of ·OH^−^ radicals, ·O^2−^ radicals, DPPH radicals, and ABTS·^+^ radicals were 3.64, 3.5, 5.07 and 5.42 mg mL^−1^, respectively (Figure 7e).

## 4 Discussion

### 4.1 Heavy Metal and Pesticide Residue Levels

*Dendrobium* flowers hold potential value both as medicinal herbs and culinary ingredients, similar to *Dendrobium* stems (Liu et al. 2020). Understanding the chemical composition, safety, and antioxidant capabilities of *D. chrysotoxum* flowers is conducive to the standardized and sustainable development of the *Dendrobium* industry. Heavy metal contamination and pesticide residues are key concerns in food safety (van Boxstael et al. 2013). Currently, research on heavy metal and pesticide residues in *Dendrobium* mainly focuses on the stems or leaves. Zhang reported that there were no health risks for adults and children from the levels of 8 heavy metals and 24 pesticide residues in the stems of *D. officinale* in Zhejiang Province (Zhang et al. 2022). Liu determined the levels of pesticide residues in 137 stems and 82 leaves of *D. officinale* in Zhejiang Province (Liu et al. 2023). It was found that multiple pesticide residues were detected in 52.56% of stem samples and 54.88% of leaf samples. However, both chronic and acute health risk assessments indicated that the pesticide residues in *D. officinale* pose negligible short‐term, long‐term, and cumulative risks to human health. Gu measured the levels of pesticide residues in 40 samples of *D. officinale* stems from eight provinces in China (Gu et al. 2021), while Xu assessed the levels of pesticide residues in samples of *D. officinale* stems or leaves from Zhejiang, Yunnan, and Guangzhou (Xu et al. 2021), and through chronic and acute health risk assessments, it was found that the pesticide residues in *D. officinale* would not pose serious health issues to the public. Yan found that the half‐life of 12 pesticides in *D. officinale* stems ranged from 0.9 to 14.4 days, and *D. officinale* stems harvested more than 42 days after pesticide application pose a low dietary risk to the population (Fu et al. 2021). This study is the first to report on the levels of heavy metals and pesticide residues in the flowers of *D. chrysotoxum*. The study collected flowers of *D. chrysotoxum* from 49 planting sites in five southern provinces of China and found that the levels of 5 heavy metals and 33 banned pesticide residues were within safe limits. As far as we know, farmers avoided applying pesticides during the flowering period of *D. chrysotoxum*, thus preventing direct contact between the flowers and pesticides. When *D. chrysotoxum* was in bloom, the pesticides in the environment were likely to have been mostly degraded, resulting in low pesticide residue levels in the flowers of *D. chrysotoxum*.

In Yunnan Province, the soil contains excessive levels of various heavy metals, leading to higher levels of heavy metals in plants with the ability to accumulate them, such as *Dendrobium*. Bao conducted a meta‐analysis and found that the total contamination of nine heavy metals in *Dendrobium* stems was 0.30 mg kg^−1^ (Huihui et al. 2023). Moreover, there is significant heterogeneity in the levels of 7 classes of heavy metal contamination in *Dendrobium* stems from different regions (Huihui et al. 2023). In our study, the levels of five heavy metals in the flowers of *D. chrysotoxum* were within safe limits. Wu reported that the sequestration of cadmium in vacuoles and other soluble parts is the main detoxification mechanism of *D. officinale* roots (Jiang et al. 2020). Perhaps, in the transport pathway of heavy metals from roots to stems, leaves, and flowers, different parts of the plant have varying degrees of immobilization of heavy metals, thereby reducing the heavy metal content in the flowers. The results of heavy metal and pesticide residue levels suggest that the flowers of *D. chrysotoxum* may be more suitable for consumption in terms of safety compared to the stems or leaves.

### 4.2 Toxicological Assessment

Food toxicology safety assessments can predict the toxicity and potential hazards of a particular food to humans (Ververis et al. 2020). In this study, results from acute oral toxicity tests indicated that *D. chrysotoxum* flowers (30.0 g kg^−1^) were nontoxic to rats when orally administered. Similarly, Fu investigated the acute oral toxicity of *D. officinale* flower powder at three doses, 2.0, 4.0, and 6.4 g kg^−1^, on pregnant rats and their offspring before birth, finding no significant adverse effects (Jianyun et al. 2020). Zhou conducted an acute oral toxicity test on mice using a dose of 12.5 g kg^−1^ of *D. officinale* stem powder (Wen et al. 2022), while Lu performed a similar test on mice using a dose of 10 g of *D. officinale* leaf powder (Lipin et al. 2022), with no significant adverse symptoms observed in the mice. The bacterial reverse mutation test has become the preferred method for screening the mutagenic effects of chemicals (Hamel et al. 2016). The mammalian erythrocyte micronucleus test is mainly used to detect the damage caused by chemical or physical factors to chromosomes or the spindle apparatus (Wen et al. 2022). The mouse spermatogonia chromosome aberration test focuses more on evaluating the mutagenicity of test substances on male reproductive cells (Wen et al. 2022). Zhou (Wen et al. 2022) and Lu (Lipin et al. 2022) conducted bacterial reverse mutation tests, mammalian erythrocyte micronucleus tests, and mouse spermatogonial chromosome aberration tests on *D. officinale* stem powder and leaf powder, finding that *D. officinale* exhibited no mutagenic effects, no induction of micronuclei in mouse bone marrow cells, and no induction of chromosome aberrations in mouse spermatogonia cells. Gu used the mouse bone marrow polychromatic erythrocyte micronucleus test and the Chinese hamster lung fibroblast chromosome aberration test to assess the genotoxicity of fresh *D. officinale* stems (Fang‐Fang et al. 2016). The results showed that fresh *D. officinale* did not exhibit genotoxicity within the tested dose range. Our study also did not find any mutagenic effect of *D. chrysotoxum* flower extract on mice. The mammalian red blood cell micronucleus test and the mouse spermatocyte chromosome aberration test conducted at doses of 1.67, 3.33, and 6.67 g kg^−1^ indicate that the extract of *D. chrysotoxum* flowers does not induce micronuclei in mouse bone marrow erythrocytes or cause chromosomal aberrations in mouse spermatocytes.

Wang conducted a 90‐day oral toxicity experiment on mice using *D. officinale* stem powder at three doses of 2.0, 4.0, and 8.0 g kg^−1^, and found no abnormalities in mouse activity and growth, with urine, hematology, and blood biochemistry values all within the normal physiological range (Yu et al. 2020). Yang evaluated a 90‐day oral toxicity study in mice using aqueous extracts of *D. Taiseed Tosnobile* stems at three doses of 0.8, 1.6, and 2.4 g kg^−1^, finding no changes in ophthalmic status, clinical condition, body weight, food consumption, or food efficiency. Although significant changes were observed in some hematological and biochemical parameters in the treatment groups receiving the oral *D. Taiseed Tosnobile* stem extracts, these values remained within the normal range and showed no dose dependency (Yang et al. 2018). In this study, we conducted a 90‐day oral toxicity experiment on mice using four doses of *D. chrysotoxum* flower at 0, 3.33, 6.67, and 10 g kg^−1^. The results showed no significant differences in growth, activity, and ophthalmic status among the different dose groups. Although certain hematological, biochemical, and urinary parameters exhibited significant differences between some dose groups, these differences remained within the normal range. Furthermore, histopathological examination results indicated that there were very few individual differences between certain dose groups, which could be considered spontaneous mild lesions in the animals, and no pathological changes related to the test substance were found. These results suggest that *D. chrysotoxum* flower does not exhibit acute toxicity, genotoxicity, or subchronic toxicity in humans within the safe dose range.

### 4.3 Chemical Composition and Antioxidant Capacity Assessment

The rise of modern pharmacology has sparked growing interest among researchers worldwide in exploring natural remedies for disease prevention. Compounds form the cornerstone of treatment approaches in traditional Chinese medicine, with researchers focusing on the rich chemical diversity found in natural plants. Notably, studies on *Dendrobium* have seen a surge in attention. A recent review summarized a total of 450 identified compounds from the *Dendrobium* genus, encompassing 45 alkaloids, 21 flavonoids, 83 bibenzyls, 79 phenanthrene compounds, 52 phenolic compounds, 25 lignan compounds, 8 fluorenone compounds, 8 coumarins, 33 polysaccharides, 52 sesquiterpenoids, 17 trace elements, and 17 other compounds (Li et al. 2023). However, among the five *Dendrobium* species documented in the “Chinese Pharmacopoeia,” all medicinal parts are stems; thus, previous studies on the chemical composition of *Dendrobium* primarily focused on its stems. However, Tang compared the chemical composition of stems, leaves, flowers, and roots of *D. officinale* and found that the polysaccharide content in stems was higher than in flowers, but the flavonoid and alkaloid contents were lower than in flowers (Wen et al. 2021). Liu found that the polysaccharide content of *D. huoshanense* followed the order: stems > flowers > roots > leaves (Liu et al. 2020). Previous reports suggest that *Dendrobium* flowers may possess similar chemical compositions and pharmacological activities to *Dendrobium* stems. Our prior research indicated that *D. huoshanense* flowers contain various functional compounds, with a high polysaccharide content of 2.53 ± 0.14 g kg^−1^ and a high soluble sugar content of 5.21 ± 0.15 g kg^−1^. In this study, we conducted a comprehensive analysis of the chemical composition of *D. huoshanense* flowers using a wide‐ranging targeted metabolomics approach, identifying a total of 2,047 metabolites that can be classified into 15 superclasses. Robustelli reported differences in the chemical composition of the essential oil from *D. huoshanense* flowers compared to our findings (Robustelli et al. 2021). These discrepancies may be attributed to variations in climatic conditions, soil characteristics, cultivation practices, growth periods, and sampling times, or they could result from changes in the processing methods that affected the chemical constituents of *D. huoshanense* flowers. By identifying specific amino acids and metabolites associated with antioxidant activity, we can better understand the mechanisms through which these flowers exert their health benefits. This knowledge not only enhances our comprehension of *D. chrysotoxum*'s potential as a functional food but also paves the way for future research into its therapeutic applications.

The antioxidant capacity of the stems, leaves, and flowers of various *Dendrobium* species has been extensively reported, including that of *D. huoshanense* flowers (Chen et al. 2021; Li et al. 2023). Our findings are consistent with previous studies, showing that the ability of *D. huoshanense* flowers to scavenge hydroxyl radicals, superoxide anions, ABTS radicals, and DPPH radicals increases with the concentration of the flower extract. The antioxidant capacity of D. *chrysotoxum* flowers may be related to their high polysaccharide content (Nie et al. 2020).

## 5 Conclusion

Through the determination of the levels of 5 heavy metals and 33 banned pesticide residues in *D. chrysotoxum* flowers, as well as food toxicology safety assessment, our study has demonstrated that *D. chrysotoxum* flowers pose no toxicity or potential hazards to humans. Additionally, a total of 2047 metabolites that can be classified into 15 superclasses were identified in *D. chrysotoxum* flowers. We have demonstrated that *D. chrysotoxum* flowers possess antioxidant capacity. Our research findings provide theoretical evidence for the potential value of *D. chrysotoxum* flowers as medicinal herbs and culinary ingredients.

## Conflicts of Interest

The authors declare no conflicts of interest.

## Supporting information


Data S1.

## Data Availability

Data available on request due to privacy/ethical restrictions.
