# Supplementary material for: Chemical Composition, Edible Safety, and Antioxidant Activity Evaluation of Flowers in a Medicinal Plant Dendrobium chrysotoxum
Source: Food Sci Nutr. 2025 Apr 18;13(4):e70067. doi: 10.1002/fsn3.70067 (PMC12006730; doi:10.1002/fsn3.70067)
Supplement: Supplementary file 1 — Data S1. [file FSN3-13-e70067-s001.docx]

Supplementary Table 1. Acute oral toxicity test results of *D. chrysotoxum* flowers extract.

| Gender | Initial weight | Weight change (g) | | Number of poisoned rats | Number of dead rats |
| --- | --- | --- | --- | --- | --- |
|  | g | 7 th day | 14 th day | Pieces | Pieces |
| Female | 185.7±5.0 | 209.0±4.1 | 229.4±13.4 | 0 | 0 |
| Male | 188.4±3.7 | 250.9±10.8 | 307.6±15.5 | 0 | 0 |

Supplementary Table 2. The urine test results of the 90-day oral toxicity test.

| Groups | Gender | Dose | Urinary Protein | Density | pH | Glucose | Occult Blood |
| --- | --- | --- | --- | --- | --- | --- | --- |
|  |  | g kg^–1^ BW^–1^ | g L^–1^ |  |  | mmol L^–1^ | CellμL^–1^ |
| Mid-term observation groups | Female | 10 | 0.03±0.07 | 1.019±0.004 | 5.9±0.20 | 0.00±0.00 | 0.00±0.00 |
|  |  | 0 | 0.06±0.08 | 1.018±0.006 | 6.0±0.40 | 0.00±0.00 | 0.00±0.00 |
|  | Male | 10 | 0.09±0.13 | 1.024±0.007 | 5.6±0.40 | 0.00±0.00 | 0.00±0.00 |
|  |  | 0 | 0.03±0.07 | 1.021±0.004 | 5.8±0.30 | 0.00±0.00 | 0.00±0.00 |
| Main test groups | Female | 10 | 0.00±0.00 | 1.012±0.003 | 6.60±0.39 | 0.00±0.00 | 1.00±3.16 |
|  |  | 6.67 | 0.00±0.00 | 1.009±0.003** | 6.25±0.35 | 0.00±0.00 | 1.00±3.16 |
|  |  | 3.33 | 0.00±0.00 | 1.013±0.004 | 6.70±0.35 | 0.00±0.00 | 1.00±3.16 |
|  |  | 0 | 0.00±0.00 | 1.014±0.002 | 6.70±0.35 | 0.00±0.00 | 2.00±4.22 |
|  | Male | 10 | 0.00±0.00 | 1.013±0.003 | 6.55±0.37 | 0.00±0.00 | 2.00±4.22 |
|  |  | 6.67 | 0.00±0.00 | 1.014±0.002 | 6.60±0.39 | 0.00±0.00 | 1.00±3.16 |
|  |  | 3.33 | 0.00±0.00 | 1.013±0.003 | 6.75±0.26 | 0.00±0.00 | 3.00±4.83 |
|  |  | 0 | 0.00±0.00 | 1.014±0.002 | 6.80±0.35 | 0.00±0.00 | 1.00±3.16 |

Supplementary Table 3. The effect of drum hammer dendrobium flower extract on organ weight in rats.

| Gender | Dose | Brain | Heart | Thymus | Adrenal | Liver | Kidney | Spleen | Testis | Uterus | Ovary | Epididymis |
| --- | --- | --- | --- | --- | --- | --- | --- | --- | --- | --- | --- | --- |
|  | g kg^–1^ BW^–1^ | g | g | g | g | g | g | g | g | g | g | g |
| Female | 10 | 1.93±0.06 | 1.03±0.10 | 0.41±0.06 | 0.08±0.01 | 8.21±0.76 | 1.93±0.23 | 0.63±0.08 | — | 0.70±0.19 | 0.11±0.03 | — |
|  | 6.67 | 1.96±0.08 | 0.98±0.08 | 0.39±0.12 | 0.08±0.01 | 8.01±0.73 | 1.82±0.16 | 0.59±0.12 | — | 0.67±0.16 | 0.11±0.02 | — |
|  | 3.33 | 1.97±0.10 | 1.01±0.05 | 0.39±0.09 | 0.08±0.01 | 8.13±0.70 | 1.84±0.17 | 0.60±0.09 | — | 0.68±0.15 | 0.12±0.03 | — |
|  | 0 | 1.99±0.07 | 1.04±0.08 | 0.47±0.09 | 0.08±0.01 | 8.48±0.92 | 1.93±0.17 | 0.66±0.10 | — | 0.67±0.11 | 0.12±0.03 | — |
| Male | 10 | 2.15±0.088 | 1.78±0.22 | 0.53±0.13 | 0.07±0.01 | 15.17±1.39 | 3.30±0.22 | 1.05±0.13 | 3.81±0.30 | — | — | 1.44±0.16 |
|  | 6.67 | 2.13±0.10 | 1.75±0.11 | 0.52±0.18 | 0.07±0.01 | 14.37±2.15 | 3.17±0.39 | 0.94±0.19 | 3.87±0.31 | — | — | 1.42±0.19 |
|  | 3.33 | 2.13±0.08 | 1.70±0.15 | 0.52±0.11 | 0.07±0.01 | 14.78±1.86 | 3.27±0.35 | 0.98±0.09 | 3.87±0.40 | — | — | 1.39±0.15 |
|  | 0 | 2.15±0.07 | 1.70±0.25 | 0.50±0.11 | 0.07±0.01 | 15.33±1.74 | 3.45±0.31 | 1.09±0.16 | 3.93±0.33 | — | — | 1.36±0.18 |

Supplementary Table 4. The effect of drum hammer dendrobium flower extract on organ weight to body weight ratio in rats.

| Gender | Dose | Brain/Body | Heart/Body | Thymus/Body | Adrenal/Body | Liver/Body | Kidney/Body | Spleen/Body | Testis/Body | Uterus/Body | Ovary/Body | Epididymis/Body |
| --- | --- | --- | --- | --- | --- | --- | --- | --- | --- | --- | --- | --- |
|  | g kg^–1^ BW^–1^ | % | % | % | % | % | % | % | % | % | % | % |
| Female | 10 | 0.63±0.05 | 0.34±0.02 | 0.14±0.02 | 0.26±0.04 | 2.68±0.13 | 0.63±0.06 | 0.20±0.01 | — | 0.23±0.07 | 0.37±0.10 | — |
|  | 6.67 | 0.65±0.06 | 0.32±0.03 | 0.13±0.04 | 0.27±0.05 | 2.65±0.20 | 0.60±0.09 | 0.19±0.03 | — | 0.22±0.06 | 0.37±0.08 | — |
|  | 3.33 | 0.66±0.09 | 0.34±0.04 | 0.13±0.02 | 0.25±0.04 | 2.69±0.16 | 0.61±0.05 | 0.20±0.04 | — | 0.23±0.06 | 0.38±0.09 | — |
|  | 0 | 0.61±0.07 | 0.32±0.02 | 0.15±0.03 | 0.25±0.04 | 2.60±0.07 | 0.59±0.04 | 0.20±0.03 | — | 0.21±0.04 | 0.37±0.09 | — |
| Male | 10 | 0.39±0.04 | 0.32±0.03 | 0.10±0.03 | 0.13±0.01 | 2.74±0.22 | 0.60±0.04 | 0.19±0.03 | 0.69±0.08 | — | — | 0.26±0.04 |
|  | 6.67 | 0.39±0.03 | 0.32±0.03 | 0.09±0.02 | 0.13±0.02 | 2.61±0.14 | 0.58±0.05 | 0.17±0.02 | 0.71±0.10 | — | — | 0.26±0.03 |
|  | 3.33 | 0.40±0.04 | 0.31±0.02 | 0.10±0.02 | 0.13±0.02 | 2.73±0.24 | 0.61±0.06 | 0.18±0.02 | 0.72±0.09 | — | — | 0.24±0.04 |
|  | 0 | 0.38±0.03 | 0.30±0.03 | 0.09±0.02 | 0.13±0.02 | 2.72±0.15 | 0.61±0.05 | 0.19±0.02 | 0.70±0.05 | — | — | 0.26±0.03 |


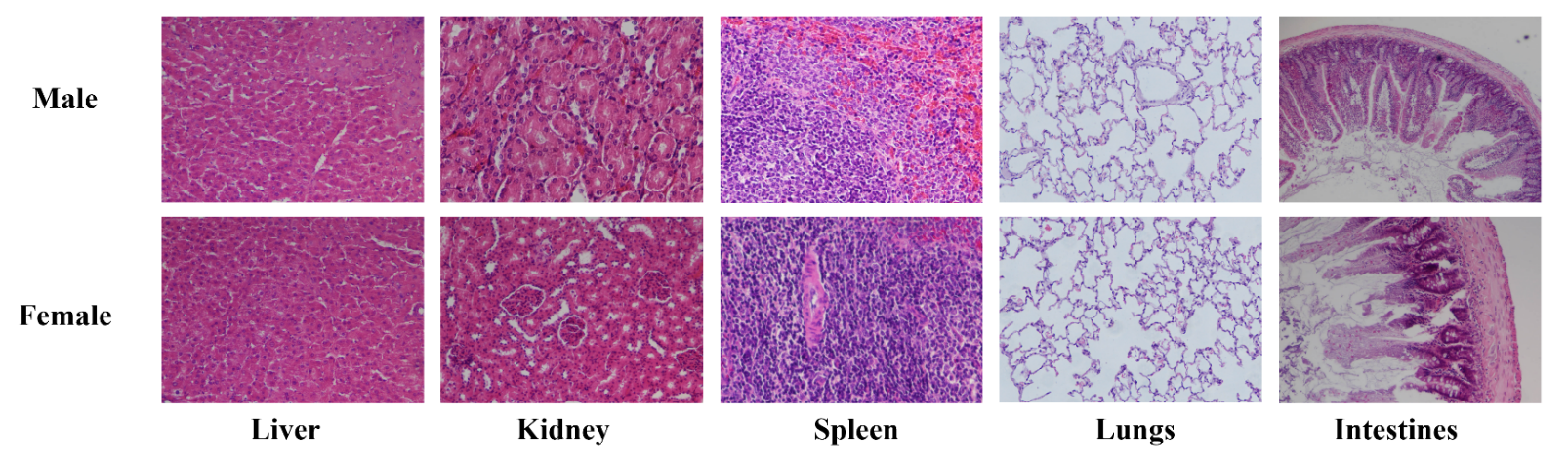


**Supplementary Figure 1. Anatomical photographs of liver, kidney, spleen, lungs and intestines of rats after the acute oral toxicity test.**


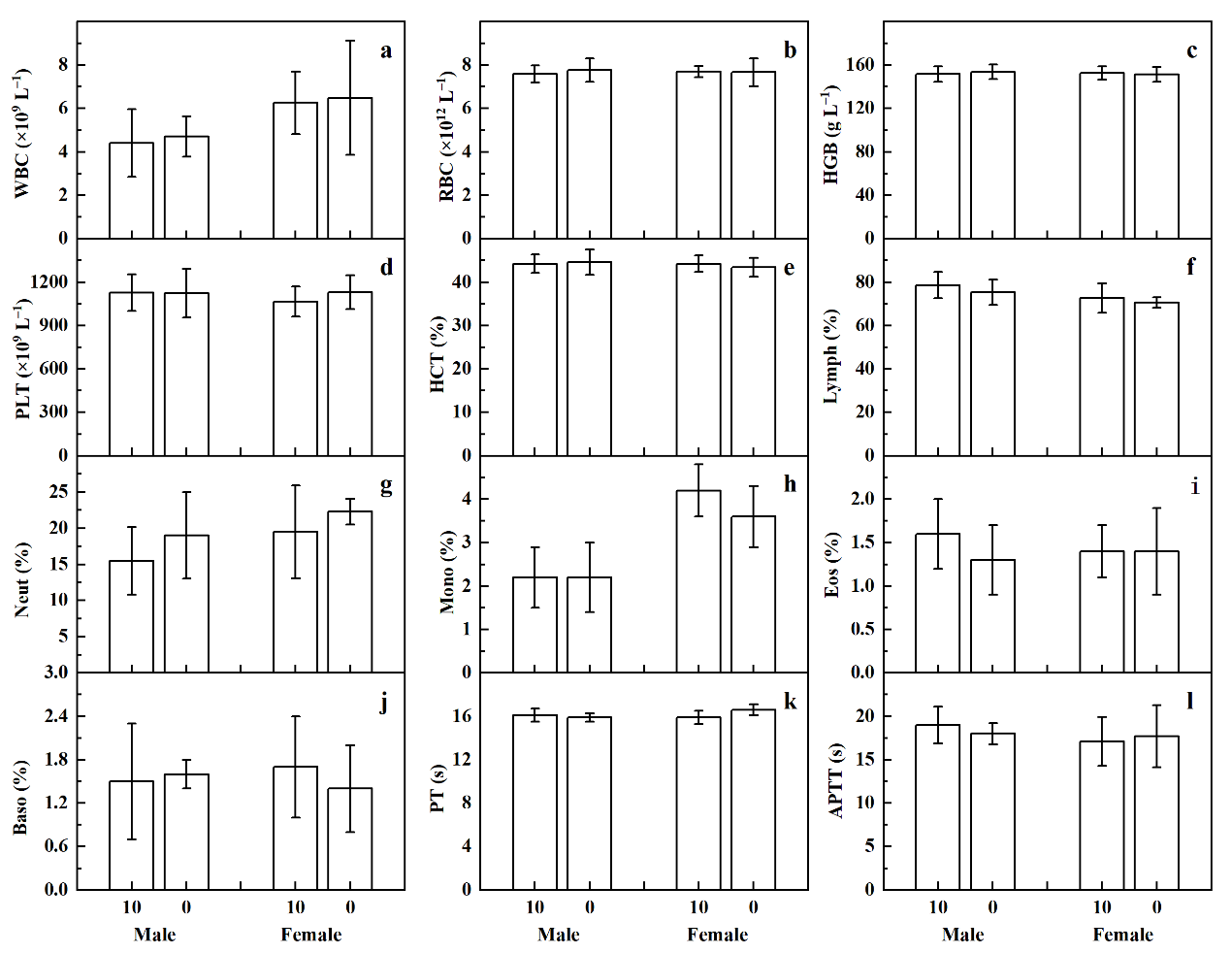


**Supplementary Figure 2. The hematological parameters of rats in the 90-day oral toxicity test of *D. chrysotoxum* flowers extract in mid-term observation groups.**

WBC stands for white blood cells, RBC stands for red blood cells, HGB stands for hemoglobin, PLT stands for platelets, HCT stands for hematocrit, Lymph stands for lymphocytes, Neut stands for neutrophils, Mono stands for monocytes, Eos stands for eosinophils, Baso stands for basophils, PT stands for prothrombin time, and APTT stands for activated partial thromboplastin time.


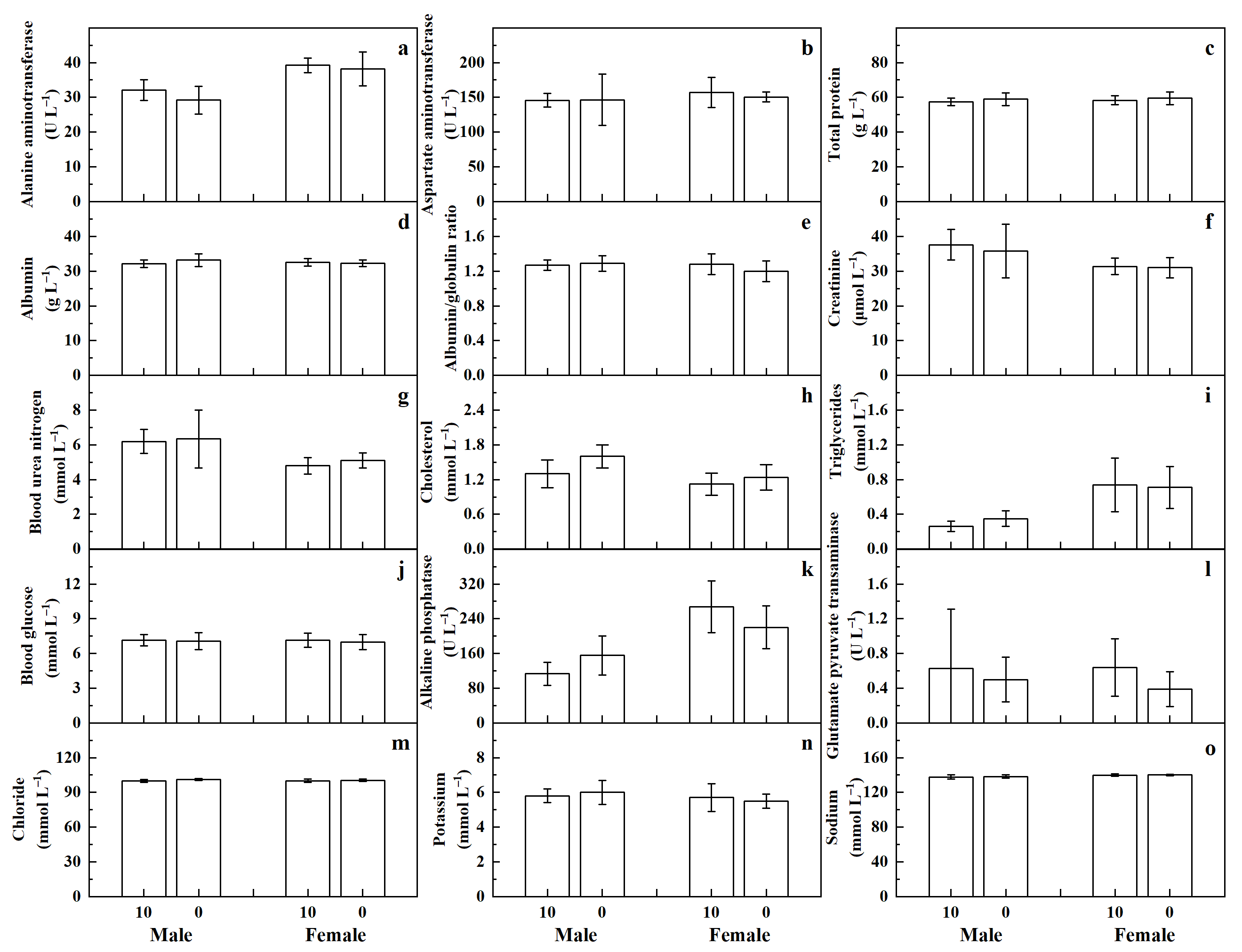


**Supplementary Figure 3. The blood biochemical parameters of rats in the 90-day oral toxicity test of *D. chrysotoxum* flowers extract in mid-term observation groups**
